# Does clinical training period support patient-centeredness perceptions of medical students?

**DOI:** 10.1080/10872981.2019.1603525

**Published:** 2019-04-15

**Authors:** Mustafa Kemal Alimoglu, Derya Alparslan, Mustafa Daloglu, Sumer Mamakli, Levent Ozgonul

**Affiliations:** a Department of Medical Education School of Medicine, Akdeniz University, Antalya, Turkey; b Emergency Service, Gökçebey State Hospital, Zonguldak, Turkey; c Department of History of Medicine and Medical Ethics, Akdeniz University, Antalya, Turkey

**Keywords:** Medical education, clinical training, hidden curriculum, patient centeredness, medical student

## Abstract

**Background**: Learning environment influences students’ professional formation and patient-centered attitudes and behaviors.

**Objective**: The purpose of this study is to investigate how hidden curriculum of learning environment and the previous experience with chronically ill patients affect patient-centeredness perceptions of medical students.

**Design**: We followed 144 students and determined their opinions on ‘ideal patient-centered practice and learning environment’ via patient-centeredness questionnaire (PCQ) just before (third year) and at the end (sixth year) of clinical training years of medical school. At the end of each clinical training year (fourth, fifth, and sixth years), we determined experiences of the students about ‘patient-centeredness of the learning environment’ using a relevant survey called communication, curriculum, and culture (C3) instrument. We also compared PCQ and C3 instrument scores of the participants who had chronically ill patient in their families/friends and who do not.

**Results**: C3 scores worsened over the years, namely, students faced increasing number of examples against patient centeredness. Final PCQ scores were worse than initial ones. C3 and PCQ scores of the students who had previous experience with chronically ill patients were not different from the scores of the remaining students.

**Conclusion**: Medical students, even those who have a chronically ill patient in their families or friends, lose their idealism about patient centeredness to some degree possibly due to hidden curriculum of the medical school.

## Introduction

In their inspiring book ‘Educating Physicians,’ Cooke et al. have identified four main goals for medical education: (1) standardization and individualization of learning process; (2) integration of basic, clinical, and social sciences, linking knowledge and medical practices and physician roles; (3) promoting habits of inquiry and improvement; and (4) formation of professional identity [1].

As one of the main goals of medical education, professional identity formation can be defined as an ongoing self-reflective process involving habits of thinking, feeling, and acting. A physician is supposed to have a deep sense of commitment and responsibility to patients, colleagues, institutions, society, and self with a desire to perform better. These habits ideally develop in ways that allow learners to demonstrate compassionate, communicative, and socially responsible physicianhood [2]. Professional identity formation occurs both intentionally and unintentionally. Besides formal education on ethics and moral reasoning or communication skills, three main aspects that shape professional identity formation are self-awareness and reflective practice, interpersonal relationships, and acculturation. Self-awareness is essential for the physician to show core values of medicine such as empathy, compassion, and altruism. Physicians should separate their own emotions and perspectives from those of the patients to communicate with compassion. They must acknowledge the patient’s needs and respond in a way that prioritizes the patient’s needs over their own beliefs, values, and emotional reactions. Such self-awareness develops through reflective process and feedback [3]. Interpersonal relationships with patients, other physicians or health professionals, communities, and system contribute to professional formation. Medical education programs allow building such relationships [4–8]. Acculturation – interaction with the values of the society, culture, or institution – also shapes medical student’s professional identity formation through a hidden curriculum. Role models in the learning environment may have positive or negative effects on the professional identity development of the medical students [9,10].

Medical schools monitor professional development of their medical students. For this purpose, direct observations, periodic meetings, and surveys or assessment tools like portfolio can be helpful to have an idea about professional behavior of a medical student [9,11–14]. Additionally, learning environment (institutional culture and hidden curriculum), which is very influential in student’s professional development, is also assessed [15].

Patient centeredness is one of the core elements of medical professionalism and an institution’s learning environment influences students’ patient-centered attitudes and behaviors [16,17]. In literature, patient centeredness of learning environment from the student perspective has already been investigated by cross-sectional studies to establish differences between medical schools or cultures [18,19]. In our study, we focused on changes in medical students’ perceptions of patient centeredness that might occur throughout medical education.

The research questions for this study were as follows:

1. Does hidden curriculum of the learning environment change patient-centeredness perception of medical students throughout clinical training years in medical school? We hypothesized that if students face negative examples in the learning environment repeatedly, they might adopt some undesired examples as normal or acceptable and their perception of ideal patient-centered practice and environment would change over time.
2. Does the presence of a chronically ill patient among family members or close friends affect patient-centeredness perceptions of medical students? We thought that the presence of a chronically ill patient among beloved persons of some students can be held as the hidden curriculum of their previous life and such students may have learned something unintentionally from experiences with these patients in favor of patient centeredness. Then, we hypothesized that there would be difference between patient-centeredness perceptions of the students who have any experience of dealing with or looking after chronically ill patients and those who do not have such experience.

The aim of the study was to investigate the effect of hidden curriculum and experience with chronically ill patients on patient-centeredness perceptions of medical students.

## Materials and methods

### Setting

As in the whole country, medical education in our school lasts 6 years. Normal biological structures and functions of human body are taught throughout the first 2 years. In this period, opportunities for medical students to observe physician–patient relationship or clinical environment are very limited unless they study with clinicians in elective social responsibility projects or special study modules. In the third year, which is a transition period between basic and clinical sciences, the students begin periodic visits to primary health-care centers or university hospital. Additionally, students study with standardized patients to learn basics of patient–physician relationship and patient-centered approach in the third year. In the next 2 years (the fourth and fifth), clinical clerkships take part. There is a 10-day medical ethics clerkship in the fourth year. The sixth (final) year is an internship period. Starting from the fourth year, the students find gradually increasing number of opportunities to observe patient centeredness of the learning environment.

### Study design

This was a prospective follow-up study of a single cohort of students. The target population was composed of medical students who were at the third year of medical education in 2012–2013 academic year. We followed this group until graduation. First, when the follow-up group was at the third year with a very limited experience of clinical environment but enough knowledge about patient centeredness, we determined their opinions on *how patient-centered practice and learning environment should be* using a questionnaire. Starting from the fourth year, every year, we determined experiences of the follow-up group about *patient-centeredness of the current learning environment* using a validated measure [20]. Finally, when the follow-up group was at the end of the sixth year, we determined their opinions on *how patient-centered practice and learning environment should be* once again to investigate if any change had occurred in time. Besides demographic characteristics, we also asked the participants if any family member or close friend had any chronic disorder thinking that there may be a difference between patient-centeredness opinions of the students who are familiar with living with patients and those who are not. A chronically ill patient was defined to the participants as the person with a chronic disease. A chronic disease is a condition that requires multiple submissions to health-care services because of its long duration and generally slow progression.

### Participants and ethical issues

We started with 213 third year medical students (89.9% of the target group). Of the remaining 26 students of the target group, 14 were missing from school on the day we delivered the questionnaires; 8 students did not return the questionnaires and 4 students did not fill in all required fields on the questionnaire properly. Through the follow-up process, we excluded those who (1) failed to pass the third year (*n* = 8), (2) failed to pass at least half of the clerkships in the fourth and fifth year (*n* = 36), and (3) did not complete at least one of the data gathering forms (*n* = 19) or completed at least one form inappropriately (*n* = 6). A total of 144 students completed all the forms properly and composed the study group. Missing 69 students were similar to the study group in terms of gender, patient-centeredness questionnaire (PCQ), and communication, curriculum and culture (C3) scores (Table 1).

**Table 1.** Comparison of study group with students lost to follow-up.

	Lost *n* = 69	Study group *n* = 144	p*	*Effect Size*
**Gender**				
**Male**	34	69	0.484^&^	0.012
**Female**	35	75		
**PCQ scores**				
**Role modeling**	30.2 ± 3.4	30.5 ± 2.5	0.180	−0.09
**Students’ experiences**	44.1 ± 4.7	44.5 ± 5.6	0.417	−0.05
Patients as objects	20.3 ± 3.6	20.5 ± 2.5	0.710	−0.01
Learning relationships	16.2 ± 2.4	16.2 ± 2.1	0.920	−0.01
Bad news for patients and students	7.8 ± 1.0	7.9 ± 1.1	0.090	−0.09
**Support for patient-centered behaviors**	11.7 ± 1.4	11.8 ± 1.6	0.627	−0.02
**Overall**	86.9 ± 7.7	86.8 ± 9.7	0.643	−0.07

*Mann–Whitney *U* test. ^&^Chi-square test.

The approval for the study was granted from Akdeniz University Board of Ethics on Noninvasive Clinical Human Studies (Reference number: 2013/324).

### Instruments

#### C3 instrument

This is a 29-item survey developed by C3 study group to quantitatively measure several characteristics of a medical school’s learning environment with respect to patient-centered care [20]. The survey should be completed by students with at least some experience in the clinical training years of medical education. The C3 instrument was validated to be used among Turkish medical students [21].

The 29-item survey has three general content areas including five dimensions:

1. ‘Role modeling’ comprises five items that measure the frequency with which students observed teachers modeling patient-centered behaviors in the course of clinical work. Each item is scored 1 (never) to 7 (always) on a 7-item Likert-type scale. Five items are scored for each of chief residents, senior residents, and interns. In total, 15 given scores are obtained in this content area. Maximum score is 105 and higher scores represent better role modeling by teachers in terms of patient-centered behaviors.
2. ‘Students’ experiences’ have three dimensions including 11 items scored 1 (very often) to 5 (never) on a 5-item Likert-type scale. This content area presents several vignettes that portray varying levels of patient-centered activities and measures how often students found themselves in similar scenarios. The first dimension is ‘patients as objects’ that includes five items inquiring frequency with which students observed the clinicians who treat the patients as an object. The second dimension is ‘learning relationships’ that includes four items inquiring how teachers or peers support the development of medical students’ skills on patient–physician relationship. The items are reverse-scored in this dimension. The third dimension is ‘bad news for patients and students’ that includes two items inquiring the frequency of negative experiences of students with conveying bad news to patients.
  - Maximum score is 55 for ‘students’ experiences’ content area and higher scores represent better patient centeredness of the learning environment.
3. ‘Support for students’ own patient-centered behaviors’ includes three items measuring how much encouragement students received when they engaged in patient-centered activities. The items are scored 1 (discouraged) to 5 (completely encouraged) on a 5-item Likert-type scale. Maximum score is 10 and higher scores represent better encouragement of students for patient-centered behaviors.

We used C3 instrument throughout the clinical training years (fourth, fifth, and sixth) to explore patient centeredness of the learning environment from the perspective of follow-up group. The reason for gathering data every year instead of once for the whole clinical training period was to follow up students’ sensitivity to recognize negative behaviors against patient centeredness such as ignoring patients’ participation in their own care or referring to patients as diagnoses, or leaving medical students alone with the patient without any support. Since no institution-wide intervention has been made for the sake of patient centeredness during the study period, we expected the students to face negative examples in the learning environment repeatedly which would lead to decreasing C3 scores year by year. Therefore, stable C3 scores over time can be interpreted in favor of students’ desensitization to recognize bad samples.

#### PCQ

We developed this questionnaire by changing the statements in the C3 instrument’s items into ‘should’ phrases. We kept the original content areas and dimensions of C3 instrument but made some minor revisions in the statements. For example, original statement ‘Please indicate how often you observed (chief residents, senior residents, or interns) communicate concern and interest in patients as unique persons’ in C3 instrument’s role modeling content area was turned to be ‘Physicians should communicate concern and interest in patients as unique persons’ in PCQ. Or the original statement ‘You hear students telling stories about patients. These stories tend to portray patients as diagnoses rather than unique human beings’ in ‘Patients as objects’ dimension of C3 was revised as ‘medical students should portray patients as unique human beings rather than diagnoses’ in PCQ.

Structure of the Likert item scales used in the C3 instrument was kept in PCQ. A 7-item Likert-type scale was used in ‘role modeling’ and 5-item Likert-type scale was used in ‘students’ experiences’ and ‘support for students’ own patient-centered behavior’ areas. Likert items were about the degree of agreement with the statement and scored 1 (absolutely not agree) to 7 (absolutely agree) in ‘role modeling’ area and 1 (absolutely not agree) to 5 (absolutely agree) in the remaining two areas. Higher scores in all area and dimensions represent higher level of idealism on patient centeredness. Maximum attainable score in the role modeling content area in PCQ was different from that of C3 instrument since each of the five items in this area was scored once in PCQ, but three times (for each of senior and junior residents and interns) in C3 instrument. Therefore, maximum score was 35 for role modeling content area in PCQ while it was 105 in C3 instrument.

We used PCQ at two points (at the end of the third year and at graduation) with the three-year interval to determine ideal patient-centeredness perceptions of the students and to explore any possible perception shift in time.

Copies of C3 instrument and PCQ were provided in Appendix.

### Data analyses

We used descriptive statistics to determine mean values. Kolmogorov–Smirnov and Shapiro–Wilk tests were used to evaluate distribution characteristics of our data. For all main variables of our study (role modeling, students experiences, support for patient-centered behaviors, and overall) *p* values for both tests were found <0.05. Additionally, for each variable, at least one of the skewness or kurtosis values (obtained by dividing statistical value by standard error) was found beyond the acceptable range of −2 and +2. Therefore, we preferred nonparametric tests. The difference between PCQ scores of the follow-up group at the start (third year of medical education) and at the end (graduation) of the study was investigated by Wilcoxon signed-rank test. We used Mann–Whitney *U* test to explore differences in C3 and PCQ scores of those who have a family member or close friend with chronic disorder and who do not. C3 scores in fourth, fifth, and sixth year were analyzed using Friedman test to explore if any change occurred in time.

*P* values of <0.05 were set for statistical signiﬁcance. Effect sizes were calculated manually using chi-square values in chi-square test and *Z* values in Mann–Whitney *U* and Wilcoxon signed-rank tests. Kendall’s coefficient of concordance was accepted as effect size for Friedman test.

## Results

Percentages of female and male participants were 52.1% and 47.9%, respectively. Mean age was 23.9 (SD 2.4) at the end of the study. Twenty-nine percent of the participants (*n* = 42, 22 female and 20 male) declared that a chronically ill patient is presented among family members or close friends.

Individual scores for each content area and dimension of the C3 instrument in clinical training years of medical education were presented in Table 1. A decline was observed in all content area and dimension scores of C3 as the students went through medical education. This decline was significant in ‘role modeling,’ ‘students’ experiences’ content areas, and in overall C3 scores with moderate effect size values (Table 2).

**Table 2.** Content area and dimension scores of the C3 instrument in clinical training years of medical education.

Content area/Dimension of C3	Student scores (mean ± SD)			p*	Effect size^β^
	Fourth year	Fifth year	Sixth year		
**Role modeling**	70.5 ± 18.1	66.3 ± 16.7	62.6 ± 14.6	<0.001	0.27
**Students’ experiences**	33.8 ± 5.5	33.0 ± 5.2	30.8 ± 4.9	<0.001	0.22
Patients as objects	13.6 ± 4.4	12.9 ± 4.2	12.0 ± 3.2	0.012	0.12
Learning relationships	12.6 ± 3.1	12.7 ± 2.6	12.0 ± 2.5	0.264	0.10
Bad news for patients and students	7.6 ± 1.4	7.4 ± 1.4	6.8 ± 1.5	0.001	0.25
**Support for patient-centered behaviors**	9.9 ± 2.9	10.6 ± 2.8	10.3 ± 2.6	0.080	0.13
**Overall**	114.2 ± 17.4	109.9 ± 17.5	103.9 ± 17.0	<0.001	0.42

*Friedman test. ^β^Kendall’s coefficient of concordance.

Final PCQ scores of the study group were found significantly lower than the initial scores with large effect size values (Table 3). Final PCQ scores were lower than initial ones also among 42 students who have a chronically ill patient among their family members or close friends. There was no difference between PCQ and C3 instrument scores of the students who are experienced with chronically ill patients and who are not (Table 4). There was no difference between initial and final PCQ scores of female and male students (Table 5).

**Table 3.** Comparison of initial and final PCQ scores representing ideal patient-centeredness perceptions of the students.

Content area/Dimension of PCQ	Student scores (mean ± SD)			
	Initial PCQ	Final PCQ	p*	Effect size
**Role modeling**	30.5 ± 2.5	24.3 ± 1.1	<0.001	−0.606
**Students’ experiences**	44.5 ± 5.6	39.4 ± 3.6	<0.001	−0.593
Patients as objects	20.5 ± 2.5	18.0 ± 2.9	<0.001	−0.541
Learning relationships	16.2 ± 2.1	14.5 ± 1.0	<0.001	−0.534
Bad news for patients and students	7.9 ± 1.1	7.0 ± 0.6	<0.001	−0.563
**Support for patient-centered behaviors**	11.8 ± 1.6	11.4 ± 1.3	<0.001	−0.412
**Overall**	86.8 ± 9.7	75.2 ± 4.4	<0.001	−0.596

*Wilcoxon signed-rank test.

**Table 4.** Comparison of the students, who have a chronically ill patient among family members or close friends, with the rest of the study group in terms of PCQ and C3 instrument scores.

Content area	Student scores (mean ± SD)			
	Patient present (*n* = 42)	Patient not present (*n* = 102)	p*	Effect size
**Role modeling**				
Initial PCQ	30.9 ± 2.6	30.4 ± 2.4	0.411	0.099
Final PCQ	24.3 ± 0.4	24.4 ± 0.6	0.259	−0.097
C3^β^	65.9 ± 15.9	64.1 ± 18.6	0.886	0.402
**Students’ experiences**				
Initial PCQ	45.5 ± 5.8	44.3 ± 5.6	0.124	0.104
Final PCQ	39.6 ± 3.6	39.4 ± 3.0	0.931	0.030
C3^β^	35.0 ± 9.6	34.5 ± 5.4	0.554	0.032
**Support for patient-centered behaviors**				
Initial PCQ	11.7 ± 1.6	11.4 ± 1.2	0.332	−0.120
Final PCQ	11.4 ± 1.4	12.1 ± 1.7	0.944	0
C3^β^	9.7 ± 2.8	10.2 ± 2.7	0.388	−0.090

*Mann–Whitney *U* test. ^β^Overall mean of fourth, fifth, and sixth year scores.

**Table 5.** Comparison of initial and final PCQ scores of the female and male students.

	Female	Male	p*	Effect size
**Initial PCQ**				
Role modeling	30.2 ± 3.4	30.8 ± 3.4	0.198	−0.10
Students’ experiences	44.6 ± 4.5	44.7 ± 4.2	0.986	−0.01
Patients as objects	20.6 ± 3.4	20.4 ± 3.0	0.547	−0.05
Learning relationships	16.2 ± 2.2	16.2 ± 2.3	0.939	−0.01
Bad news for patients and students	7.8 ± 1.1	8.1 ± 1.2	0.173	−0.11
Support for patient-centered behaviors	11.9 ± 1.1	11.8 ± 1.3	0.744	−0.02
Overall	86.5 ± 5.5	87.5 ± 6.0	0.344	−0.08
**Final PCQ**				
Role modeling	24.2 ± 1.9	24.3 ± 1.6	0.235	−0.09
Students’ experiences	39.7 ± 5.3	39.5 ± 3.9	0.971	
Patients as objects	17.8 ± 2.7	18.2 ± 2.5	0.461	−0.06
Learning relationships	14.8 ± 4.1	14.2 ± 2.0	0.353	−0.07
Bad news for patients and students	7.0 ± 1.0	7.0 ± 1.0	0.730	−0.03
Support for patient-centered behaviors	11.4 ± 1.1	11.3 ± 1.3	0.384	−0.07
Overall	75.0 ± 5.6	75.2 ± 4.2	0.658	−0.036

*Mann–Whitney *U* test.

## Discussion

This study aimed to investigate the effect of hidden curriculum and experience with chronically ill patients on patient-centeredness perceptions of medical students. Data analyses provided three main results. First, C3 scores decreased, namely, students faced increasing number of negative examples in terms of patient centeredness of the learning environment throughout clinical training years of medical education. In other words, the hidden curriculum of the learning environment worked against patient centeredness moderately according to effect size estimates (Table 2). The decrease in C3 scores is an expected finding since the probability of observing undesired behaviors increases as time goes by even if the number of such behaviors remained constant. In the current study, if no change was found in C3 scores which represented observation frequency of misbehaviors in clinical setting, then we would think that our students adopted some behaviors against patient centeredness and started to ignore some of them. However, on the basis of our results, it is clear that our students have not lost their sensitivity to recognize poor examples throughout clinical training years. Second, main result of this study indicates that the students, even those who have a chronically ill patient in their families or friends, lose ideal patient-centeredness notions to some degree at graduation possibly thinking that ideal practice and environment might be less patient-centered compared to that they conceived 3 years ago. Large effect size estimates point out that it is highly possible for clinical training to play a major role on idealism shift of our students (Table 3). In our opinion, for a medical student, losing idealism to some degree but not losing sensitivity or ability to recognize bad samples against patient centeredness is a critical but reversible point. Therefore, we plan some refresher training activities like conferences and discussion sessions in our school for recall of idealism in our students’ minds.

We could not find any study in the literature that has specifically focused on the shift in patient-centeredness perceptions of medical students. However, there are studies reporting erosion in moral reasoning or ethical principles throughout undergraduate and postgraduate medical education [22,23]. These studies suggest that observations of unethical and unprofessional conduct are major contributors to moral erosion among medical students. We believe that the reason for the shift in our students’ perceptions of patient centeredness is the same, i.e., observations of wrong role models and misbehaviors lead a perception shift toward less patient centeredness in medical students’ minds. Literature suggests that physicians trained in patient centeredness tend to be more compassionate, humanistic, and relate better with patients [24,25]. Therefore, a patient-centered learning environment in a medical school is crucial.

In our school of medicine, we try to give extracurricular messages to our students on professionalism and patient centeredness starting from very early periods of medical education. For example, we organize a white coat ceremony for the first year students at the beginning of each academic year. In this ceremony, speakers emphasize the importance of professional values and patient-centered approaches. Another example is career counseling meetings, in which career pathways in different branches of medicine are presented by outstanding professionals. These professionals also emphasize the importance of professionalism and patient centeredness in their talks. Additionally, our curriculum includes courses on humanities, ethics and professionalism, relevant practical sessions with simulated and real patients, etc. Literature suggests that such curricular changes do not produce the desired results when all efforts had been made to the learner side alone [24,26–28]. We totally agree with the literature and believe that patient centeredness of a learning environment should also be supported by faculty, residents, and staff. However, concerns about cost-effectiveness, the fragmentation of medicine subspecialties, and technological developments have left patient-centered care behind in today’s world [29–31]. Goldstein et al. suggest that creating a safe environment in which an aspirational approach to professionalism is addressed throughout the entire institutional culture. Such an environment faculty and residents must visibly and consistently demonstrate patient-centered approach in their practice. In addition, when students or others observe or experience unprofessional behaviors in the institutional setting, there must be effective mechanisms for bringing these to institutional attention. Such mechanisms must ensure action, accountability, and confidentiality [32].

Third, main finding of the current study was that the presence of a chronically ill patient among family members or close friends did not make any difference in students’ opinions of an ideal patient-centered environment or observation frequencies of misbehaviors in clinical training years. Actually, our expectation was in favor of a difference at least in the final PCQ results. The reason why we expected a difference was the thought that if a beloved person around a student has any disease and needs to be in close relationship with health-care teams, then such a student may have already observed or heard about some good or undesired behaviors of caregivers and developed insight especially about rights and uniqueness of a patient. When these experiences and insights are reinforced with formal education in medical school, our expectation was that these students would have a sound perception of ideal patient centeredness and never compromise on their idealism after clinical training despite bad role models or misbehaviors. This hypothesis was refuted, namely, perceptions of patient centeredness of those who have a chronically ill patient among family or friends shifted toward less patient centeredness just like the perceptions of the rest of the study group. Actually, for this special group of students, we talk about two hidden curricula: the first one is the hidden curriculum of their previous life experiences with chronically ill patients which we expected to affect their perceptions of patient centeredness positively and the second one is the hidden curriculum of clinical training years in medical school which we expected to affect perceptions of patient centeredness negatively. It seems that the second one is powerful enough to eliminate the effect of the first one over time. Therefore, institutions, administrators, and medical educators should make every effort to improve patient centeredness of learning environments in medical schools to cope with negative effects of hidden curriculum.

Our study has two major limitations. First, our results that were obtained from only one medical school with a limited number of students cannot be generalized. Second, we did not have any qualitative data which would help better understand the reasons behind the results. For example, we accepted that the presence of experiences with a chronically ill patient near the student was enough to make him/her experienced about patient centeredness to some degree. However, there may be a chronically ill patient in family, but the student may not have enough experience with that person in terms of patient centeredness. On the other hand, there may be no chronically ill patient around, but a student may have had information about patient centeredness. We did not ask any question to the students about level and frequency of experiences with the patients. In order to clarify such conditions, open-ended questions in the text format or focus group interviews would be helpful.

In conclusion, throughout clinical training years of medical education, medical students even those who have a chronically ill patient in their families or friends seem to lose their idealism to some degree about patient-centered practice and environment. Hidden curriculum, i.e., bad role models or misbehaviors observed by the students in clinical settings, may be responsible from perception shift toward less patient centeredness. Larger studies supported additionally by qualitative findings with larger populations in various medical schools are needed to have more insight about the effect of hidden curriculum on patient-centeredness perceptions of medical students.

## Appendix Data gathering tools

**Table:**

C3 instrument	PCQ
**Content area 1: Role modeling**	**Content area 1: Role modeling**
1–3. Please indicate how often you observed (chief residents, senior residents or interns) communicate concern and interest in patients as unique persons.	1. Physicians should communicate concern and interest in patients as unique persons.
4–6. Please indicate how often you observed (chief residents, senior residents or interns) encourage patients’ participation in their own care.	2. Physicians should encourage patients’ participation in their own care.
7–9. Please indicate how often you observed (chief residents, senior residents or interns) take seriously patients’ concerns about their conditions or care.	3. Physicians should take seriously patients’ concerns about their conditions or care.
10–12. Please indicate how often you observed (chief residents, senior residents, or interns) develop good rapport with patients.	4. Physicians should develop good rapport with patients.
13–15. Please indicate how often you observed (chief residents, senior residents, or interns) explore emotional aspects of patients’ illnesses.	5. Physicians should explore emotional aspects of patients’ illnesses.
**Content area 2: Students’ experiences**	**Content area 2: Students’ experiences**
‘Patients as objects’ dimension	‘Patients as objects’ dimension
1. You overhear an attending physician discussing a patient’s case history with another attending or house officer. During the course of the conversation, the patient is referred to as a diagnosis.	1. Physicians should *not* refer to a patient as diagnoses.
2. When you describe social history information about a patient (e.g., career, hobbies) during ward rounds, you notice that the rest of the team is not paying attention.	2. Health-care teams should pay attention to social history information about a patient (e.g., career, hobbies) as much as medical history.
3. During rounds, your attending is paged to his office. His secretary has a patient present and wants to know when the attending will return. The attending replies, ‘Tell the patient to wait. I’ll get there when I get there’.	3. Physicians should take care of his/her appointments with patients and respect to patients’ time.
4. You hear students telling stories about patients. These stories tend to portray patients as diagnoses rather than unique human beings.	4. Medical students should portray the patients as unique persons rather than medical diagnoses.
5. Your ward team is rounding on a patient in his room when one of the consulting services arrives for this patient. Your attending and the consulting attending proceed to talk about the patient’s case as if the patient weren’t there.	5. When two or more physicians talk about a patient’s case in the same environment with the patient, they should respect to presence of the patient.
‘Learning relationships’ dimension 1. You hear students telling stories about patients. These stories tend to portray how the relationship with the patient affected the student(s) personally.	‘Learning relationships’ dimension 1. Medical students should be aware that the relationship with the patient affects the student(s) personally.
2. During your third or fourth year of medical school, an attending or house officer observes you while you interview a patient and provides you with feedback on your bedside manner.	2. Medical educators should observe students while they interview a patient and provide them with feedback.
3. During your third or fourth year of medical school, you are asked to interview a patient (either ‘real’ or ‘standardized’) and you are provided with feedback on how well you listened to the patient (either from the patient or an observer).	3. Interviews with patients (either ‘real’ or ‘standardized’) should take part within the medical education curriculum.
4. You are given advice from students in the classes ahead of you on what you need to do to succeed in medical school. This advice emphasizes the importance of good communication skills with patients.	4. Medical students should appreciate the importance of good communication skills with patients as a key for success in medical school.
‘Bad news for patients and students’ dimension	‘Bad news for patients and students’ dimension
1. During a rotation in the outpatient clinic, you have to convey bad news to a patient without any teaching or discussion about how to break the news in a caring manner.	1. Medical students should *not* have to convey bad news to a patient without any teaching or discussion about how to break the news in a caring manner.
2. You and your ward team have to convey bad news to a patient. Sometime after the bad news is conveyed, you find yourself having to answer many of the patient’s questions about the news without any teaching about how to talk to patients after they have been given bad news.	2. After the bad news is conveyed, medical students should *not* find themselves having to answer many of the patient’s questions about the news without any teaching about how to talk to patients after they have been given bad news.
**Content area 3: Support for students’ own patient-centered behaviors**	**Content area 3: Support for students’ own patient-centered behaviors**
1. In general, when I made an effort to develop rapport with patients, my instructors … me.	1. Instructors should encourage medical students when they made an effort to develop rapport with patients.
2. In general, when I made an effort to get to know patients as unique persons, my instructors … me.	2. Instructors should encourage medical students when they made an effort to get to know patients as unique persons.
3. In general, when I made an effort to legitimize patients’ concerns about their condition or care, my instructors … me.	3. Instructors should encourage medical students when they made an effort to legitimize patients’ concerns about their condition or care.

PCQ: Patient-centeredness questionnaire. *The C3 instrument was originally developed by communication, curriculum and culture (C3) study group (ref. no: 20).
